# Physical activity around the clock: objectively measured activity patterns in young children of the GECKO Drenthe cohort

**DOI:** 10.1186/s12889-019-7926-3

**Published:** 2019-12-05

**Authors:** Rikstje Wiersma, Congchao Lu, Esther Hartman, Eva Corpeleijn

**Affiliations:** 1 Department of Epidemiology (HPC FA40), University Medical Center Groningen, University of Groningen, PO Box 30.001, 9700 RB Groningen, The Netherlands; 2 Center for Human Movement Sciences, University of Groningen, University Medical Center Groningen, Section F, PO Box 196, 9700 AD Groningen, The Netherlands; 3 School of Public Health, Tianjin Key Laboratory of Environment, Nutrition and Public Health, Centre for International Collaborative Research on Environment, Nutrition and Public Health, Tianjin Medical University, Tianjin, China

**Keywords:** Sedentary time, Moderate-to-vigorous physical activity, Accelerometry, Preschool children, Obesity

## Abstract

**Background:**

Given the widespread problem of physical inactivity, and the continued growth in prevalence of childhood and adolescent obesity, promotion of regular physical activity (PA) among young people has become a public priority. A greater understanding of children’s PA patterns throughout the day is needed to effectively encourage children to be more physically active. Hence this study looking at the distribution of PA in young children throughout the day and its relevance to overweight.

**Methods:**

Accelerometers (ActiGraph GT3X, weartime > 600 min/day, ≥3 days) were used to measure the PA of 958 children (aged 5.7 ± 0.8 years, 52% boys) enrolled in the GECKO Drenthe cohort. Levels of sedentary time (ST), light PA (LPA) and moderate-to-vigorous PA (MVPA) were recorded throughout the day and analysed in segments (07:00–09:00, 09:00–12:00, 12:00–15:00, 15:00–18:00, 18:00–21:00). Body mass index was measured by Preventive Child Healthcare nurses and Cole’s (2012) definition of overweight was used. General linear mixed models, adjusted for age, sex and season, were used to analyse patterns of PA and ST throughout the day.

**Results:**

Children were most sedentary in the early morning (07:00–09:00) and evening (18:00–21:00), and exhibited the most time spent engaged in LPA and MVPA in the afternoon (12:00–15:00) and late afternoon (15:00–18:00). The greatest inter-individual variation in ST, LPA and MVPA among the children occurred in the late afternoon and evening (approximately 40, 30 and 15 min difference per time segment between 25th and 75th percentile, respectively). The most active children (highest quartile of MVPA) were found to be more active and less sedentary throughout the entire day than the least active children (lowest quartile of MVPA). Furthermore, children with overweight were no less active than children without overweight.

**Conclusions:**

At this young age, the relevance of different PA patterns to childhood overweight was minimal. Children were most active in the afternoon and late afternoon. To encourage PA in general, ST can be reduced and PA increased in the early morning and evening. Targeted PA interventions to specifically stimulate the least active children could take place in the late afternoon or evening.

## Background

Physical activity (PA) is an important factor in human health. Individuals who are physically active have a lower risk of developing diseases [1, 2]. Furthermore, PA is considered to be a key component in the prevention and management of overweight and obesity [3–5].

Several different organizations have provided guidelines for PA in children and young people [6–11]. It is recommended that preschool children (< 5 years old) engage in at least 180 min of activity each day. At least 60 min of this PA should be of moderate-to-vigorous intensity. Furthermore, children should not remain seated or sedentary for periods of more than 1 h at a time [6, 7]. It is recommended that primary school children and young people (in the 5–17 age group) should engage in at least 60 min of moderate-to-vigorous physical activity (MVPA) per day. They should also minimize the time spent in extended periods of sedentary activity [8–11]. Nevertheless, many children are not complying with these PA guidelines. As a result, they may be at greater risk of developing overweight, obesity and other health problems. Since the development of overweight starts at a young age, it is useful to try and support higher levels of PA early in life [12].

Most studies into children’s PA behaviours are performed in children of school age. However, a large study using data from the International Children’s Accelerometry Database (ICAD) showed that time spent in PA during childhood slightly increases from 2 to 5 years and then decreases progressively over time until the age of 18 [13]. An opposite pattern for sedentary time (ST) was observed [13]. Accordingly, opportunities to boost PA levels of children around the age of 5 should be examined. Furthermore, children’s PA behaviours are often analysed on the basis of an average daily PA. The use of accelerometer data makes it possible to analyse PA patterns in greater detail. A recent study revealed differences in the distribution of MVPA levels during the day, even between children with the same average PA levels [14]. In Europe, there have been no previous studies of PA patterns in young children, and of their relevance to health outcomes such as overweight.

The PA behaviour of older children throughout the day has been more extensively studied. One accelerometry-based study in school-age children (7–11 years old) found that children’s PA behaviour was more consistent in the school environment (07:00–15:00), whereas the greatest variation in PA levels occurred in the early evening (17:00–19:00) [15]. One study into highly active and low active children (aged 10–11) found that, in four of the five time segments, the former achieved significantly more moderate PA and vigorous PA than the latter [16].

The aim of our study was to identify segments of the day with potential for targeted PA interventions in young children. We explored patterns of different intensities of objectively measured PA and ST throughout the day to examine when young children were more sedentary and when they were more active. Secondly, since PA is an important factor in the prevention of overweight and obesity, we examined the association between the PA distribution throughout the day and childhood overweight.

## Methods

### Study design

The GECKO (Groningen Expert Center for Kids with Obesity) Drenthe study is a population-based birth cohort focusing on early risk factors for overweight and obesity. Details of the GECKO Drenthe cohort are described elsewhere [17]. In 2006, almost 3000 pregnant women were recruited. The children involved are currently being monitored and have been since the last trimester of their mother’s pregnancy. Written informed parental consent was obtained for participation in the study, also for any minors to take part in the study. The study was approved by the Medical Ethics Committee of the University Medical Center Groningen (UMCG), in accordance with the 1975 Declaration of Helsinki (as revised in 1983). The study has been registered at www.birthcohorts.net.

### Measurements and data analysis

#### Physical activity

PA data was collected from May 2011 to October 2013. PA was measured using ActiGraph GT3X accelerometers (ActiGraph, Pensacola, FL). These devices have been shown to be appropriate and reliable for the measurement of PA volume and intensity in young children [18, 19]. Children wore an ActiGraph (held in place by an elastic belt) on their right hip throughout their waking hours on four consecutive days, except while bathing or swimming. Data was collected at a frequency of 30 Hz and was analysed at a 15-s epoch recording. Non-wearing time was defined as periods of at least 90 min with zero counts [20]. Those days in which the weartime amounted to less than 600 min were excluded from the analyses. Subsequently, any children who had fewer than three valid wear days were excluded from the analyses. When sent by post, some accelerometers generated a valid wearing day (> 10 h/day). These ‘postage days’ were identified by low-light activity (≤ 100 min/day) and deleted. Outcome measures were assessed using the following cut-off points: ST (≤ 819 cpm), light physical activity (LPA) (820–3907 cpm) and MVPA (≥ 3908 cpm) [21]. These cut-off points were the best fit for our age group.

Five time segments were used for the purpose of studying the distribution of MVPA and ST throughout the day. These time segments were in accordance with Dutch school schedules. They were defined as ‘early morning’ (07:00–09:00), ‘morning’ (09:00–12:00), ‘afternoon’ (12:00–15:00), ‘late afternoon’ (15:00–18:00) and ‘evening’ (18:00–21:00). All children in this study were attending kindergarten (referred to as ‘Group 1’ or ‘Group 2’ in the Dutch educational system). Here, children are given structured educational instructions – in the context of play – and they have ample opportunity to move around freely. Although the age range for preschoolers in most studies is between 2 and 5 years, we have defined the children in this study as ‘preschoolers’ because their behaviour is likely to be comparable with preschoolers. The activity levels per segment were expressed as the average number of minutes of activity per hour. Any incomplete hours were excluded. The cumulative activity levels were calculated as minutes per day (06:00–23:00).

To study PA patterns in general, we pooled the results for all the children in the study. In addition, to specifically stimulate the least active children, we examined differences between the most active and the least active children. Based on children’s daily MVPA, the children were grouped into sex-specific quartiles, ranging from least active (Q1) to most active (Q4). Alternatively, children could be classified as ‘active’ if they achieved the MVPA exercise standard (≥ 60 min of MVPA per day) on more than 50% of their valid days [16, 22]. If the average MVPA is used instead of this method of only counting days when the children were compliant with the standard, only 9.6% of the children would be reclassified. In view of this small difference, plus the need to maximise distinctiveness and variations in the number of valid days from one child to another, the decision was taken to use quartiles of average daily MVPA.

#### Weight status and additional data collection

Height and weight were measured by trained Preventive Child Healthcare nurses, according to a standardized protocol. Children were weighed while wearing light clothing, on an electronic scale with a digital read-out. Their weight was recorded to the nearest 0.1 kg. Their height was measured using a stadiometer and recorded to the nearest 0.1 cm. Each child’s body mass index (BMI, kg/m^2^) was converted into age- and sex-specific standardized BMI Z-scores. This involved the use of Dutch growth analysis software (Growth Analyzer 3.5; Dutch Growth Research Foundation, Rotterdam, The Netherlands), using 1997 population data as the reference [23]. Individuals were classified either as not affected by overweight (underweight and normal weight) or affected by overweight (overweight and obesity), using age- and sex-specific cut-off points for children, based on Cole et al. 2012 [24]. Questionnaires completed during pregnancy were used to obtain details of the parents’ educational level (low/middle education or higher vocational education) and of the total household income. With regard to the seasons, winter was defined as December – February, spring as March – May, summer as June – August and autumn as September – November.

### Statistical analysis

The statistical analysis was performed using IBM SPSS Statistics (version 23). All MVPA variables were log-transformed, as they were not normally distributed. Independent t-tests and χ^2^-tests were used to check for differences between children with and without valid PA data. Repeated measures ANOVA tests were performed – with the segments as within-subjects variables – to justify the selected time segments and to determine whether it would be useful to include an extra segment around midday (12:00–13:00). General linear mixed models were used, for each segment and for the whole day (cumulative), to examine any differences in PA and ST patterns between active and less active children and between children with and without overweight. In each of the general linear mixed models used, the participant number was entered as subject and activity group (most active vs. least active) or weight status (overweight, yes/no) was entered as fixed factor. To control for possible differences due to sex, age or season of PA measurement, we entered sex, age and season as fixed factors as well. With regard to random factors, the intercept was included and the participant number was entered. In addition, the whole-day analyses were adjusted for accelerometer weartime. The significance level was set at *p* < 0.05.

## Results

**Fig. 1:**
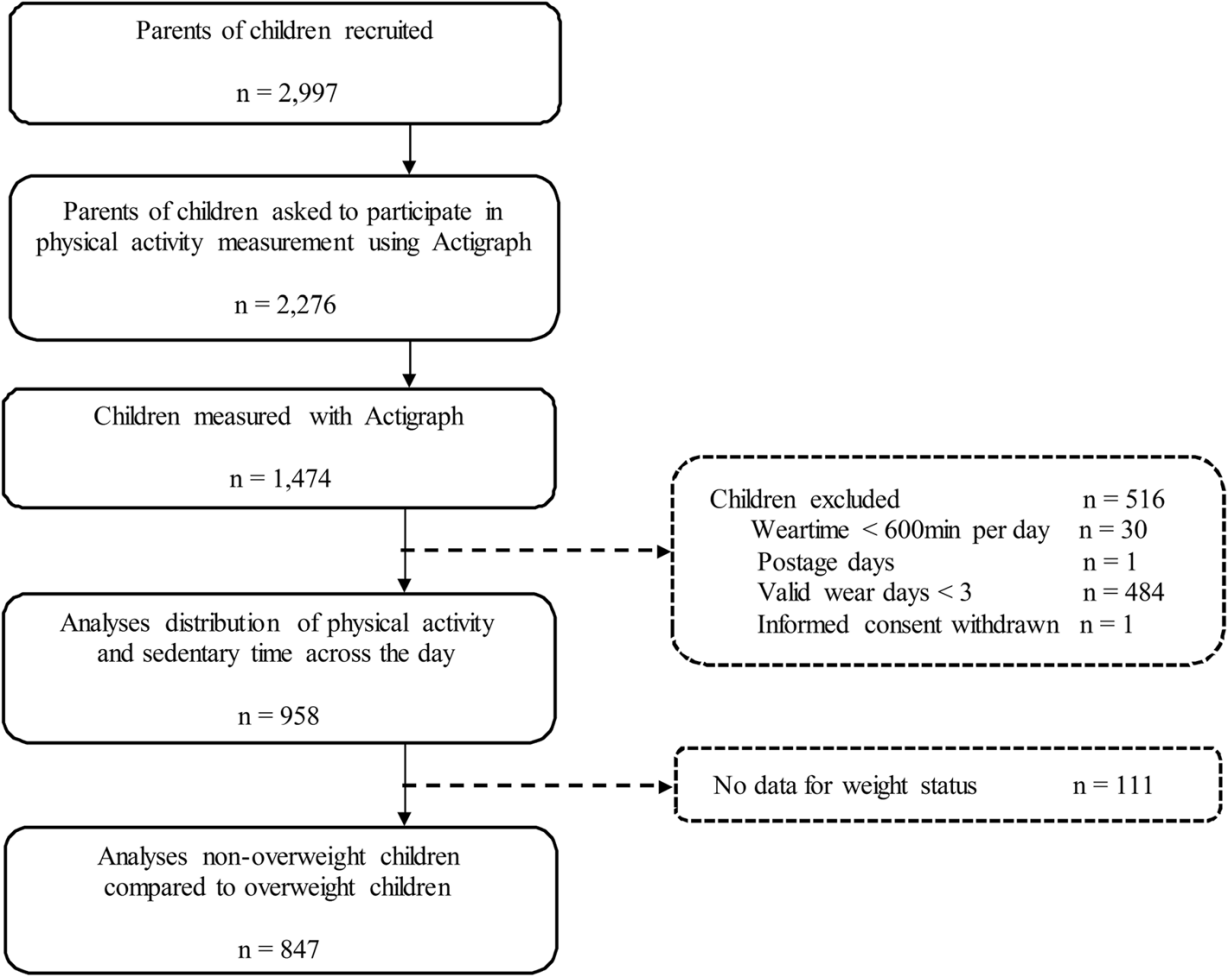
Flowchart showing participant recruitment in GECKO Drenthe cohort

In total, the parents of 2276 children were asked to participate by allowing their child’s PA to be measured. As a result, the PA of 1474 children was measured using ActiGraph accelerometers (response rate = 64.8%; for flow chart see Fig. 1). The final sample consisted of 958 children with valid accelerometer data. The majority of these children were between 4 and 6 years of age (5th – 95th age percentile: age 4.4–7.0). For 847 of these children, weight status data was also available.

The characteristics of the children and their parents are shown in Table 1. Approximately 46% of the children met the PA guideline of at least 60 min per day in MVPA. There were no significant differences between children with and without valid PA data, in terms of their descriptive characteristics, except for the educational level of the father. The fathers of children with valid PA data tended to be more highly educated (41.8%) than the fathers of children without valid PA data (31.6%) (*p* < 0.001). The descriptive characteristics of children in the most active and least active groups and those of children with and without overweight are shown in Table 5 in the Appendix. The least active children (Q1) were significantly younger than the most active children (Q4) (aged 5.5 and 5.8, respectively). In addition, the least active children (Q1) wore their device for a shorter period than the most active children (654 and 663 min/day, respectively). With regard to the children with and without overweight, the fathers of children without overweight tended to be more highly educated than the fathers of children affected by overweight.

**Table 1:** Child characteristics

	*N*	*mean ± SD or %*
Sex (boys)	500	52.2%
Age PA measurement (years)	958	5.7 ± 0.8
Age BMI measurement (years)	847	5.8 ± 0.3
BMI (kg/m^2^)	847	15.9 ± 1.3
BMI Z-score	847	0.20 ± 0.78
Weight status		
Underweight	54	6.4%
Normal weight	722	85.2%
Overweight	61	7.2%
Obese	10	1.2%
Ethnicity (Dutch)	865	90.6%
Education level (low/middle)		
Mother	432	51.6%
Father	428	54.5%
Household income		
< €1150	21	2.3%
€1151–€3050	508	55.3%
€3051–€3500	163	17.8%
> €3501	124	13.5%
Unknown /not reported	102	11.1%
Weartime (min/day)	958	655.9 ± 35.3

*Abbreviations*: *PA* physical activity, *BMI* body mass index

### Distribution of physical activity and sedentary time throughout the day

Firstly, the validity of the selected time segments was assessed. For ST, LPA and MVPA, main effects were found between segments throughout the day (all *p* < 0.001). With certain exceptions, the contrasts showed significant differences between all segments (*p* < 0.001). The exceptions were between 12:00–15:00 and 15:00–18:00 for ST (*p* = 0.504), between 09:00–12:00 and 15:00–18:00 for LPA (*p* = 0.196) and between 09:00–12:00 and 18:00–21:00 for MVPA (*p* = 0.106). This means that most segments differed significantly from each other and that they can be regarded as separate time slots throughout the day. The option of an extra segment around midday (12:00–13:00) was explored and discarded, as it was not considered to be useful. In the case of ST and MVPA, no significant differences were found between the midday period and the 13:00–15:00 segment. In the case of LPA, there was only a small difference.

#### Sedentary time

**Fig. 2:**
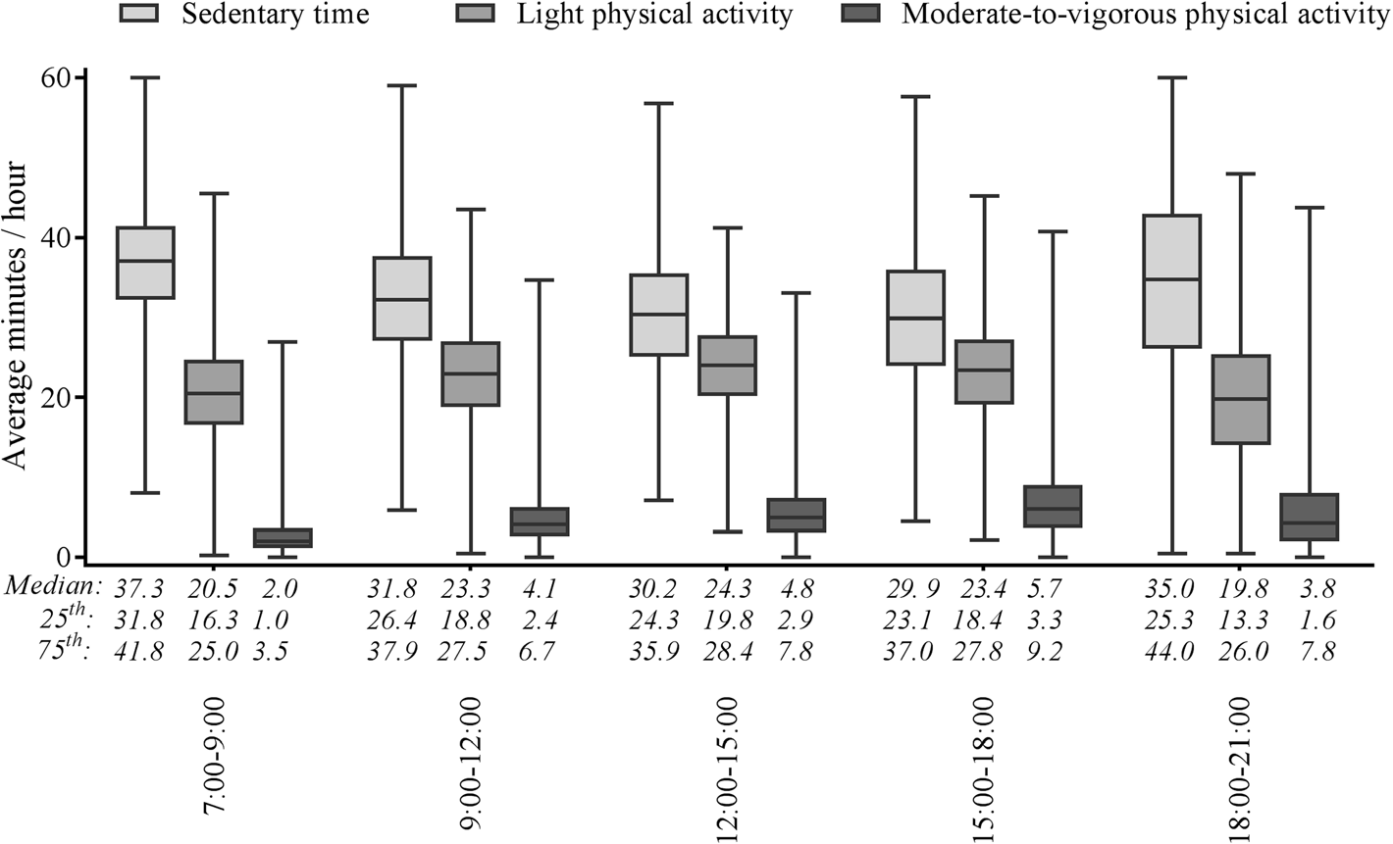
The flow of PA levels throughout the day. Sedentary time (ST), light physical activity (LPA) and moderate-to-vigorous physical activity (MVPA) per time segment with median, 25th – 75th percentile and minimum and maximum. For clarification, the exact numbers are given below the x-axis. Almost all time segments showed significant differences. The exceptions were for ST between 12:00–15:00 and 15:00–18:00, for LPA between 09:00–12:00 and 15:00–18:00 and for MVPA between 09:00–12:00 and 18:00–21:00

Table 2 indicates the pattern of PA throughout the day. It gives details of the average time spent sedentary, in LPA and in MVPA for all segments throughout the day. Children are most sedentary during the early morning (07:00–09:00) and evening (18:00–21:00). Figure 2 shows the median, the 25th – 75th percentile and the minimum and maximum values for ST, LPA and MVPA per segment, throughout the day. This highlights the changes in PA levels throughout the day, as well as differences between the children. In terms of ST, it shows that the greatest differences between children occur in the evening (18:00–21:00). In this time segment, 25% of all children spent up to 44 min per hour in sedentary activity. However, the lowest 25% were only sedentary for 25 min per hour. Over the entire 3 h of this time segment, the most sedentary 25% of children exhibited ST for about 130 min, while the corresponding value for the least sedentary 25% of children was 75 min.

**Table 2:** Time spent sedentary, in light physical activity and in moderate-to-vigorous physical activity throughout the day

Segment	N	Sedentary time	Light PA	Moderate-to-vigorous PA
7:00–09:00	928	36.7 ± 6.3 (61.2%)	20.6 ± 5.3 (34.4%)	2.7 ± 2.0 (4.4%)
9:00–12:00	958	32.2 ± 6.2 (53.6%)	22.9 ± 4.8 (38.2%)	4.9 ± 2.5 (8.1%)
12:00–15:00	958	30.2 ± 5.5 (50.3%)	24.0 ± 4.1 (40.1%)	5.7 ± 2.7 (9.6%)
15:00–18:00	958	30.1 ± 6.3 (50.2%)	23.1 ± 4.4 (38.6%)	6.7 ± 3.2 (11.2%)
18:00–21:00	903	34.4 ± 9.6 (57.3%)	19.7 ± 6.5 (32.9%)	5.9 ± 4.9 (9.9%)
Cumulative	958	346.2 ± 55.0 (52.8%)	248.9 ± 37.3 (37.9%)	60.8 ± 24.0 (9.3%)

Data for sedentary time, light physical activity and moderate-to-vigorous physical activity are presented as mean ± SD (%) in average minutes per hour (time segments) or per day (cumulative)

#### Physical activity

The PA pattern in Table 2 shows that children spent most time engaged in LPA and MVPA in the afternoon (12:00–15:00) and late afternoon (15:00–18:00), respectively. In addition, Fig. 2 shows that the greatest differences between children, in terms of LPA and MVPA, occur in the late afternoon and evening (18:00–21:00). Take the average MVPA level in the late afternoon, for instance. This derives from the fact that 25% of the entire group of children spent a total of approximately 30 min in MVPA in the period from 15:00 to 18:00, while the least active 25% of the children spent less than 10 min in MVPA during this time segment.

**Fig. 3:**
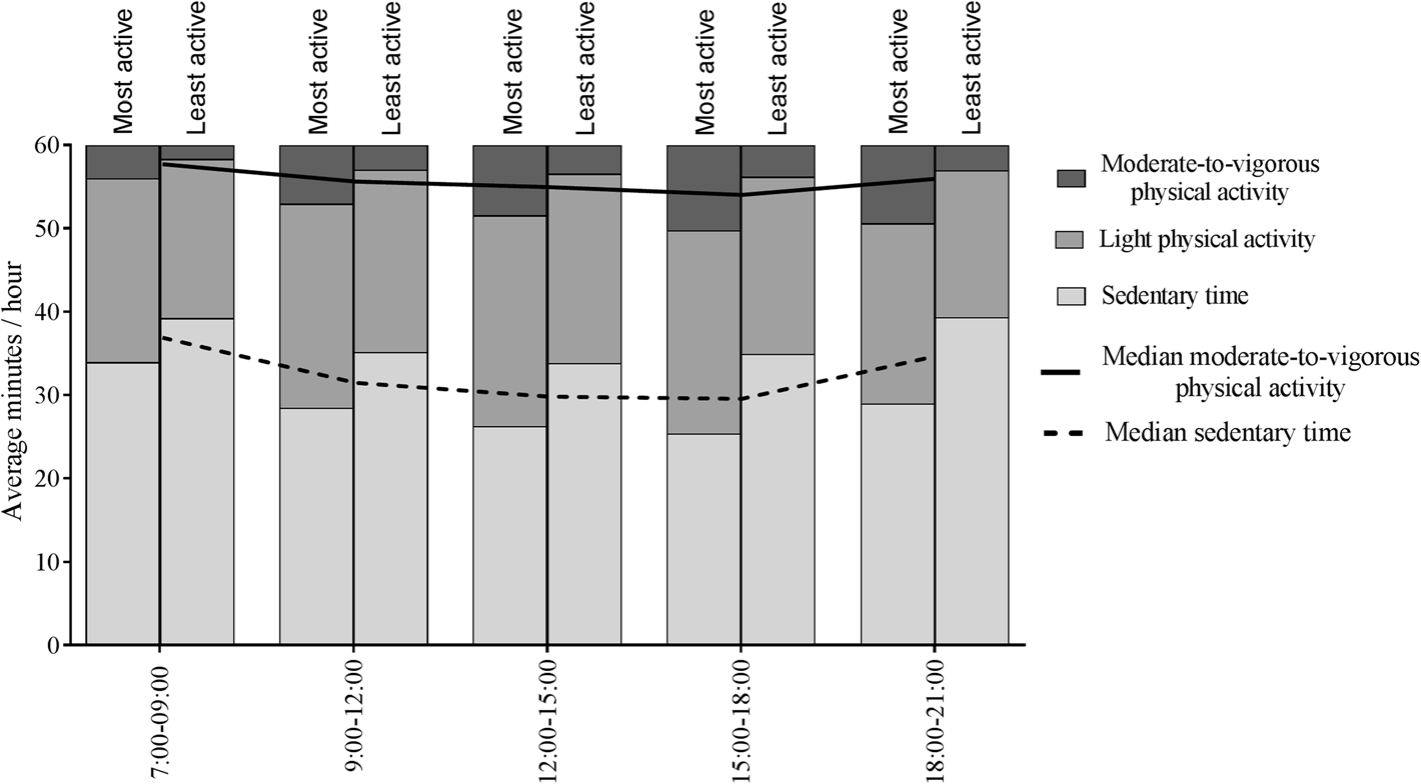
Daily physical activity patterns in the most active and least active children. Sedentary time, light physical activity and moderate-to-vigorous physical activity per time segment for the most active and least active children. For comparison, the lines represent the median sedentary time and moderate-to-vigorous physical activity of the entire group of children. All time segments throughout the day showed significant differences between the most active and least active children, adjusted for sex, age at physical activity measurement and season

Further looking into the differences between the most active children and the least active children, the former were more active and less sedentary than the latter throughout the entire day. Significant differences were found between the activity groups for ST, LPA and MVPA in every time segment of the day. With regard to the average time spent in ST, LPA and MVPA, the largest differences between the most active and least active children occurred in the late afternoon and the evening (Table 3; Fig. 3).

**Table 3:** Active children are more active throughout the day

Segment	Most active (Q4) (mean minutes / hour)	Least active (Q1) (mean minutes / hour)	Estimate [95%CI] (minutes / hour)
Sedentary time (ST)			
ST 7:00–09:00	33.9 [33.1; 34.7]	39.4 [38.7; 40.2]	5.6 [4.5; 6.7]
ST 9:00–12:00	28.2 [27.5; 28.8]	35.5 [34.8; 36.2]	7.3 [6.4; 8.3]
ST 12:00–15:00	25.9 [25.3; 26.5]	34.1 [33.5; 34.8]	8.2 [7.4; 9.1]
ST 15:00–18:00	25.5 [24.9; 26.2]	35.1 [34.4; 35.7]	9.5 [8.6; 10.4]
ST 18:00–21:00	29.7 [28.6; 30.8]	39.7 [38.6; 40.8]	10.0 [8.5; 11.5]
Cumulative ST (per day)^a^	301.8 [297.1; 306.4]	393.0 [388.5; 397.6]	91.3 [84.7; 97.9]
Light physical activity (LPA)			
LPA 7:00–09:00	22.2 [21.6; 22.9]	18.9 [18.3; 19.6]	−3.3 [−4.3; −2.4]
LPA 9:00–12:00	24.8 [24.2; 25.3]	21.6 [21.0; 22.1]	−3.2 [− 4.0; − 2.4]
LPA 12:00–15:00	25.6 [25.1; 26.1]	22.5 [22.0; 23.0]	− 3.1 [− 3.8; − 2.4]
LPA 15:00–18:00	24.3 [23.8; 24.9]	21.2 [20.7; 21.7]	− 3.2 [− 3.9; − 2.4]
LPA 18:00–21:00	21.3 [20.5; 22.1]	17.3 [16.5; 18.1]	− 4.0 [−5.2; − 2.9]
Cumulative LPA (per day)^a^	266.5 [262.3; 270.7]	230.9 [226.8; 235.0]	−35.6 [− 41.5; − 29.7]
Moderate-to-vigorous physical activity (MVPA)^b^			
MVPA 7:00–09:00	3.4 (2.0–4.9)	1.4 (0.9–2.3)	−0.8 [−0.9; − 0.7]
MVPA 9:00–12:00	6.8 (4.8–8.8)	2.9 (2.2–3.7)	− 0.9 [−1.0; − 0.8]
MVPA 12:00–15:00	8.0 (6.7–10.1)	3.3 (2.5–4.3)	−1.0 [− 1.1; − 0.9]
MVPA 15:00–18:00	9.5 (7.9–12.0)	3.6 (2.8–4.6)	−1.1 [− 1.2; − 1.0]
MVPA 18:00–21:00	8.1 (5.1–12.0)	2.3 (1.4–3.8)	−1.1 [− 1.2; − 0.9]
Cumulative MVPA (per day)^a^	89.8 (79.4–100.9)	35.2 (29.0–41.8)	−1.0 [− 1.0; − 0.9]

Descriptives of ST and LPA are presented as adjusted means and 95% confidence intervals. Descriptives of MVPA are presented as medians with 25th and 75th percentiles. Analyses were performed using linear mixed models, adjusted for sex, age at PA measurement and season. All *p*-values were < 0.001

^a^ Analyses additionally adjusted for accelerometer weartime

^b^ Statistical testing for MVPA was performed using log-transformed MVPA

### The relevance of PA distribution throughout the day to overweight

**Fig. 4:**
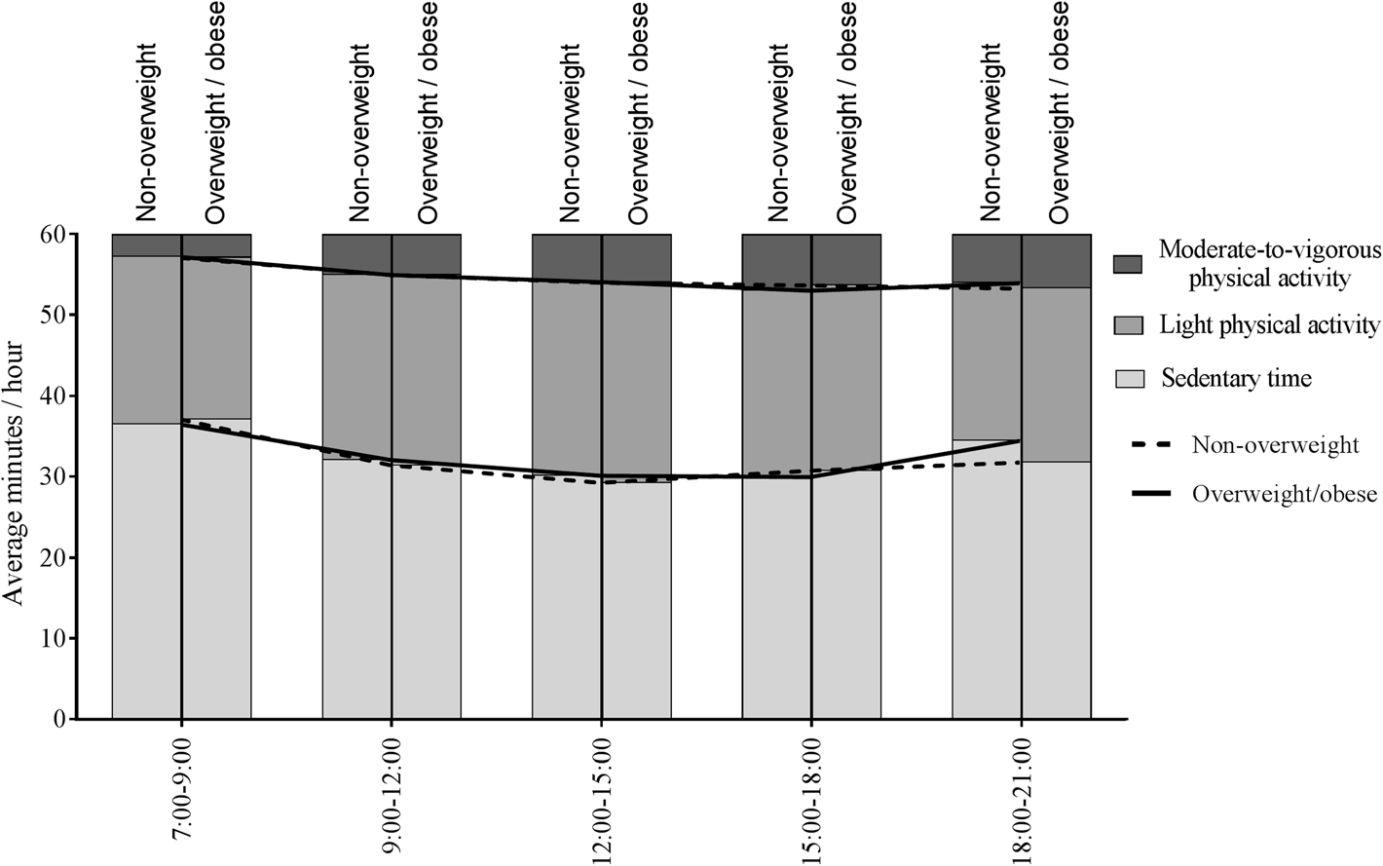
Daily physical activity patterns in children with and without overweight. Sedentary time, light physical activity and moderate-to-vigorous physical activity per time segment for children with and without overweight, with means per behaviour and per group. There were significant differences between children with (n = 71) and without overweight (n = 776) in terms of sedentary time and light physical activity, but only in the evening (18:00–21:00). All analyses were adjusted for sex, age at physical activity and body mass index measurement and season

In the evening, marginal differences between children with and without overweight were found with regard to ST and LPA, but not in terms of MVPA. Children affected by overweight exhibited less ST and more LPA in the evening (18:00–21:00) than children without overweight (Table 4; Fig. 4).

**Table 4:** PA patterns throughout the day for children with and without overweight

Segment	Overweight (mean minutes / hour)	Non-overweight (mean minutes / hour)	Estimate [95%CI] (minutes / hour)
Sedentary time (ST)			
ST 7:00–09:00	37.1 [35.6; 38.5]	36.6 [36.2; 37.1]	0.4 [−1.1; 2.0]
ST 9:00–12:00	31.6 [30.3; 32.9]	32.2 [31.8; 32.6]	−0.6 [−2.0; 0.7]
ST 12:00–15:00	29.4 [28.2; 30.7]	30.3 [29.9; 30.7]	−0.9 [−2.2; 0.4]
ST 15:00–18:00	31.2 [29.7; 32.6]	30.4 [29.9; 30.8]	0.8 [−0.7; 2.3]
ST 18:00–21:00	32.6 [30.5; 34.7]	35.1 [34.4; 35.8]	**−2.5 [−4.7; −0.4]**
Cumulative ST (per day)^a^	343.8 [332.8; 354.7]	348.6 [345.2; 351.9]	−4.8 [−16.2; 6.6]
Light physical activity (LPA)			
LPA 7:00–09:00	20.1 [18.8; 21.3]	20.7 [20.4; 21.1]	−0.7 [−1.9; 0.6]
LPA 9:00–12:00	23.5 [22.5; 24.5]	22.9 [22.6; 23.2]	0.6 [−0.4; 1.6]
LPA 12:00–15:00	24.7 [23.8; 25.6]	24.0 [23.7; 24.3]	0.7 [−0.2; 1.7]
LPA 15:00–18:00	22.7 [21.7; 23.7]	22.9 [22.6; 23.2]	−0.2 [−1.2; 0.8]
LPA 18:00–21:00	21.1 [19.7; 22.5]	19.2 [18.8; 19.7]	**1.8 [0.4; 3.3]**
Cumulative LPA (per day)^a^	252.5 [244.6; 260.3]	247.7 [245.3; 250.1]	4.8 [− 3.4; 13.0]
Moderate-to-vigorous physical activity (MVPA)^b^			
MVPA 7:00–09:00	2.5 (1.4–3.8)	2.1 (1.3–3.4)	0.1 [−0.1; 0.2]
MVPA 9:00–12:00	4.7 (3.1–6.3)	4.4 (3.1–6.2)	−0.01 [− 0.1; 0.1]
MVPA 12:00–15:00	5.2 (3.6–7.6)	5.4 (3.8–7.2)	0.02 [− 0.1; 0.1]
MVPA 15:00–18:00	5.5 (3.6–8.4)	6.4 (4.4–8.7)	−0.1 [− 0.2; 0.03]
MVPA 18:00–21:00	5.3 (3.2–8.6)	4.5 (2.5–8.0)	0.2 [− 0.01; 0.4]
Cumulative MVPA (per day)^a^	56.7 (44.2–73.8)	57.5 (44.0–75.0)	−0.01 [− 0.1; 0.1]

Descriptives of ST and LPA are presented as adjusted means and 95% confidence intervals. Descriptives of MVPA are presented as medians with 25th and 75th percentiles. Analyses were performed using linear mixed models, adjusted for sex, age at PA measurement, age at BMI measurement and season. The bold values were statistically significant (*p* < 0.05)

^a^ Analyses additionally adjusted for accelerometer weartime

^b^ Statistical testing for MVPA was performed using log-transformed MVPA

## Discussion

This study shows that young children’s PA and ST exhibit a distinct pattern throughout the course of the day. The highest level of ST occurred in the early morning (07:00–09:00), while the highest levels of LPA and MVPA occurred in the afternoon (12:00–15:00) and late afternoon (15:00–18:00). In terms of ST, LPA and MVPA, the largest differences between children were found to occur in the late afternoon and evening. Active children were more active and less sedentary throughout the day, rather than being more active in one particular segment. Contrary to what might be expected, no clear association was found between PA patterns and overweight. Children affected by overweight exhibited less sedentary time and more light PA in the evening than children without overweight, but the differences were marginal.

The early morning (07:00–09:00) was the first time segment identified for targeted interventions to boost PA levels in children, since all of the children were relatively sedentary during this period. However, there is some doubt about the scope for boosting PA during this segment, as most children follow a standard routine of waking up, having breakfast and getting ready for school. Nevertheless, if PA could be integrated into this routine (e.g. by the inclusion of an active commute to school), this could be a sustainable way of structurally increasing daily PA levels. This strategy is promoted by the Comprehensive School Physical Activity Program (CSPAP), which aims to exploit all opportunities for children to be more physically active before, during and after school [25]. Potentially, the difference between the 25th and 75th percentile is 2.5 min of MVPA per hour and 8.7 min of LPA. This means that more than 10 min per hour can be devoted to PA instead of ST. Thus, there is indeed scope for improving PA levels in the early morning, especially when such measures can be incorporated into children’s morning routines.

Two other time segments with potential for targeted PA interventions were the late afternoon (15:00–18:00) and evening (18:00–21:00). With regard to MVPA, both the median and the 75th percentile were higher in the late afternoon (Fig. 2). During this 3 h period, the 25th and 75th percentiles of MVPA differed by approximately 18 min and the differences in terms of LPA and ST were approximately 30 and 40 min, respectively. Another study that looked at hourly activity patterns over the day in 4-year-old Swedish children, showed a slightly different pattern [26]. They showed that children were more active and less sedentary from 9:00 to 15:00 (during preschool) compared to the rest of the day [26]. They assessed PA using the same type of accelerometer device and the same accelerometer cut-offs. However, they did not explore the variation in activity between children. The observed differences between the 25% most active and the 25% least active children in the current study showed significant opportunities for increasing the PA levels of the least active children in the late afternoon. During the evening segment (18:00–21:00) the differences between the 25th and 75th percentile were even greater – almost 1 h for ST and 19 min for MVPA. These substantial differences suggest considerable variation in the range of activity exhibited by children before they go to sleep. Some children seem to prefer active forms of relaxation, whereas other children appear to favour more passive forms (ST). Indeed, studies in older children have found that the variation in activity is greater in the late afternoon and evening. A study in 7- to 11-year-old children in northwest England found the greatest variation in PA behaviour in the early evening (17:00–19:00) [15]. Another study, into highly active and low active 10- to 11-year-old children suggested the use of structured PA programmes to promote PA in low active children during the after-school time segment (15:30–18:30) [16]. Furthermore, although the morning and late afternoon/evening seem to offer the best opportunities for changing PA levels, PA can also be promoted during school hours. PA during school time may even enhance academic engagement and performance [27, 28]. The current study shows how different types of interventions may be suitable at different moments of the day, based on an analysis of young children’s natural PA patterns. The children’s PA patterns show the need for interventions to increase PA levels in children, as only half of the children met the PA guideline of at least 60 min per day in MVPA. Tailored PA interventions are needed to determine whether inactive children can be stimulated to become more active and to determine which types of interventions are most effective in preschool children.

There were no substantial differences between children with and without overweight in terms of their cumulative PA and ST throughout the day. However, when the day was broken down into individual time segments, we found that, during the evening segment, children affected by overweight exhibited significantly less ST and more LPA than children without overweight. Although, in absolute terms, the differences were marginal, it was clear that the children with overweight were no less active than the children without overweight. This finding may however depend on the context in which children live. In Chinese preschool children, a study using accelerometers to assess PA, comparable to ours, showed that, during some time segments, children affected by overweight were less active compared to children without overweight [29]. The Chinese children with overweight or obesity were more sedentary from 17:00 to 18:00 and spent less time in LPA from 8:00 to 11:00 and from 17:00 to 21:00 on schooldays compared to children without overweight. A possible explanation is that the Chinese school schedule includes an obligatory nap time from 12:00 to 14:00 which probably influences, and maybe even suppresses, children’s natural activity patterns across the day [29]. This was visible when looking at the average MVPA level of the Chinese children, which was 47 min per day during schooldays and 61 min per day during weekends, the latter being comparable to the average of 61 min MVPA per day in our study. Our finding that children with overweight are no less active than children without overweight may be explained by the weartime. Children affected by overweight may have a longer weartime because they sleep less. A previous study showed a lack of sleep to be a determinant of overweight in young children [30], so it may be worthwhile to establish whether or not this might have been a factor in the present study. An analysis of the time length for which children wore their accelerometers revealed that while this was not significantly longer for children with overweight, this group did tend to stay up a little longer than their peers. Another possible explanation concerns the use of BMI to classify overweight, as BMI is not an exclusive measure of fat mass [31, 32]. A higher BMI could indicate a higher muscle mass rather than a higher fat mass, resulting in relatively muscular children being classified as overweight [31, 32]. A recent review and meta-analyses examining the association between PA and adiposity in young children showed no association between PA and BMI, irrespective of the intensity of PA [33]. However, the review and meta-analyses did show that young children with overweight spent less time in MVPA compared to children without overweight [33]. Nevertheless, the influence of MVPA on weight status was rather small. A large study in 2015 also found no differences in PA levels between two to six-year-old children with and without overweight [13]. Only in older children, from age seven onwards, children affected by overweight were found to be less active than children without overweight [13]. Yet, even if the effect of PA does increase with age, it is still important to intervene at a relatively young age. The prevention of overweight at a young age is more effective than treatment after its onset [34]. Also, low levels of PA at a young age may make children more prone to develop overweight as they grow older, as young children’s PA behaviours are likely to track into later life [35]. In summary, our results provided no evidence to suggest that PA patterns across the day are relevant to young children’s weight status. It is worth remembering that PA at a young age is also important in terms of other health issues (e.g. motor development, fitness and bone and skeletal health) [1, 2]. Therefore, it is recommended that further research is carried out into the influence of PA behaviours throughout the day on other health outcomes.

### Study limitations and strengths

To the best of our knowledge, this is one of only a small number of studies into varying intensities of objectively measured PA, at different times of the day, in preschool children. The use of accelerometry makes it possible to define patterns throughout the day and to analyse them in more detail. This provides a basis for more specific recommendations concerning ways in which PA levels could be improved in preschool children. One strength of the study is that the participants of the GECKO Drenthe cohort form a highly representative sample of the general population, including children from families of both high- and low socioeconomic status. Furthermore, about half of the children who were born in Drenthe between April 2006 and April 2007 were included in the GECKO Drenthe cohort. One limitation that should be mentioned is that there may have been differences in the time children spent at school and the time at which their school day ended. However, the selected time segments were found to be valid, so any effect it might have had on the results was considered negligible. We performed a sensitivity analysis, taking into account only those days in which we were certain that children were at school. The results were the same.

## Conclusion

At this age, the relevance of different PA patterns to childhood overweight was minimal. To encourage PA in general, ST can be reduced and PA increased in the early morning and evening. Children were most active in the late afternoon. Targeted PA interventions to specifically stimulate the least active children could take place in the late afternoon or evening. This is based on the large inter-individual variation observed during these particular time segments.

## Data Availability

The datasets used and/or analyzed during the current study are available from the corresponding author on reasonable request.

## Appendix

**Table 5:** Descriptive characteristics of the most active and least active children and of the children with and without overweight

	Most active (*n* = 239)	Least active (*n* = 239)	Overweight (*n* = 71)	Non-overweight (*n* = 776)
Sex (boys)	125 (52.3%)	125 (52.3%)	32 (45.1%)	402 (51.8%)
Age PA measurement (yrs.)^a^	5.8 ± 0.8	5.5 ± 0.8	5.7 ± 0.8	5.7 ± 0.8
Age BMI measurement (yrs.)	5.8 ± 0.3	5.8 ± 0.4	5.9 ± 0.4	5.8 ± 0.3
BMI (kg/m^2^)^b^	15.9 ± 1.3	15.9 ± 1.4	18.9 ± 1.0	15.7 ± 1.0
BMI Z-score^b^	0.21 ± 0.78	0.17 ± 0.80	1.73 ± 0.36	0.06 ± 0.65
Weight status				
Underweight	16 (7.5%)	13 (6.2%)	–	–
Normal weight	180 (84.1%)	182 (86.3%)	–	–
Overweight	17 (7.9%)	11 (5.2%)	–	–
Obese	1 (0.5%)	5 (2.4%)	–	–
Ethnicity (Dutch)	209 (87.8%)	217 (91.2%)	61 (85.9%)	704 (91.1%)
Education level (low/middle)				
Mother	113 (54.6%)	97 (46.9%)	33 (57.9%)	346 (50.4%)
Father^b^	104 (55.0%)	96 (47.3%)	38 (69.1%)	341 (53.3%)
Household income				
< €1150	10 (4.4%)	3 (1.3%)	1 (1.4%)	20 (2.7%)
€1151–€3050	126 (55.5%)	132 (56.7%)	41 (59.4%)	405 (54.5%)
€3051–€3500	46 (20.3%)	41 (17.6%)	7 (10.1%)	135 (18.2%)
> €3501	24 (10.6%)	31 (13.3%)	7 (10.1%)	105 (14.1%)
Unknown /not reported	21 (9.3%)	26 (11.2%)	13 (18.8%)	78 (10.5%)
Weartime (min/day)^a^	662.8 ± 36.5	654.0 ± 34.6	662.2 ± 39.7	655.2 ± 34.6

Children were divided into activity groups based on sex-specific quartiles of their average MVPA per day

Children were classified either as affected by overweight/obesity or not affected by overweight based on the definition used by Cole et al. (2012)

Abbreviations: *PA* physical activity, *BMI* body mass index. Data are presented as mean ± SD or N (%)

^a^Significant differences between most active and least active children

^b^ Significant differences between children with and without overweight or obesity

## Abbreviations

BMI: Body mass index
CSPAP: Comprehensive School Physical Activity Program
GECKO: Groningen Expert Center for Kids with Obesity
LPA: Light physical activity
MVPA: Moderate-to-vigorous physical activity
PA: Physical activity
ST: Sedentary time
UMCG: University Medical Center Groningen
